# Characterizing the burden of premature ejaculation from a patient and partner perspective: a multi-country qualitative analysis

**DOI:** 10.1186/1477-7525-6-33

**Published:** 2008-05-12

**Authors:** Dennis Revicki, Kellee Howard, Jennifer Hanlon, Sally Mannix, Alison Greene, Margaret Rothman

**Affiliations:** 1 Center for Health Outcomes Research, United BioSource Corporation, Bethesda, Maryland, USA; 2 QualityMetric, Lincoln, Rhode Island, USA; 3 Johnson & Johnson Pharmaceuticals, Raritan, New Jersey, USA

## Abstract

**Background:**

Premature ejaculation (PE) is a common sexual dysfunction among men which affects men and their partners. Little qualitative data are available to characterize the impact of PE on men and their partners about ejaculatory control, sexual satisfaction, emotional distress and relationships. The objective of this study was to assess the impact of PE from the perspective of men with PE and the female partners of men with PE on their sexual experience, distress and relationships.

**Methods:**

Qualitative data were collected through 14 focus groups in the US and through one-on-one interviews in the US, UK, Italy, France, Germany, and Poland. Study participants included heterosexual men with PE and female partners of males with PE. All participants were asked about how PE affects their daily life, including emotional impacts. One-on-one interviews also included obtaining feedback on the male and female versions of 4-single item measures of PE focusing on ejaculatory control, satisfaction with intercourse, interpersonal distress, and relationship difficulty.

**Results:**

Participants included 172 males with PE and 67 female partners of men with PE. Lack of control over ejaculation and dissatisfaction with intercourse emerged as central themes of PE. Lack of ejaculatory control resulted in greater dissatisfaction and greater emotional distress, including feelings of inadequacy, disappointment, and anxiety. Continued PE ultimately leads to greater problems with partners and often disrupts partner relationships. Participants indicated that PE was keeping them from attaining complete intimacy in their relationships even when their partners were generally satisfied with sexual intercourse. Impacts of PE on sexual satisfaction, emotional distress and partner relationships were consistent across countries. Feedback on the single-item PE measures confirmed relevance of the item content and further confirmed major themes identified from the qualitative data.

**Conclusion:**

This qualitative study provides valuable insights on the substantial psychosocial burden of PE in the US and the Europe. Lack of control over ejaculation resulted in dissatisfaction with intercourse and increased emotional distress, and wide-ranging impact for both men with PE and their partners of men with PE. Data collected in this study may help inform the content of new patient reported measures for use in PE research.

## Introduction

Research on the understanding and assessment of premature ejaculation (PE) has increased over the past five years [1]. Although there are varied conceptualizations of PE, the most widely accepted definition is included in the most recent version of the Diagnostic and Statistical Manual of Mental Disorders (DSM-IV-TR) [2,3]. The DSM-IV-TR defines PE as "persistent or recurrent onset of orgasm and ejaculation with minimal sexual stimulation before, on, or shortly after penetration and before the person wishes it" that "cause(s) marked distress or interpersonal difficulty." Differences in definitions of PE persist and there is debate about the role of patient-reported outcomes (PROs) in determining the diagnosis of PE [4-6]. The current study focused on examining patient perceptions of the key problems and burden associated with PE from the perspective of men with PE and female partners of men with PE.

PE is the most frequent male sexual dysfunction with an estimated 20% to 30% of men reporting PE (variously defined) at some time in their life [7-11]. Despite this prevalence rate, few men receive effective medical or psychological treatment for PE, although men have reported self-treatment with a variety of behavioral approaches, creams, or herbal products [12]. Failure to seek treatment may be attributable to personal sensitivity about the problem, failure of men or their primary care physicians to discuss sexual issues, and perceptions that there are no effective interventions.

Intravaginal ejaculatory latency time (IELT) is the most frequently used endpoint in clinical trials of PE treatments [2,4,5,13,14]. However, IELT measures only one component of the disorder. Some researchers suggest that there is little empirical support for including patient perceptions of control over ejaculation or psychological distress in addition to IELT for diagnosing PE or assessing treatment outcomes in PE [4,5]. However, other researchers have found that patient perception may play a significant role in assessing the burden of PE [1,6,15].

Previous research has indicated that patient-reported outcomes (PROs) are important for PE assessment and should measure different aspects of the disorder, including perception of ejaculatory control, satisfaction with ejaculatory control, and satisfaction with sexual intercourse [1,6,15-18]. For example, Rosen et al. found that a combination of IELT and patient-reported control over ejaculation, satisfaction with intercourse, personal distress and interpersonal difficulty best predicted PE diagnostic status [6]. Patrick et al. found that IELT had a direct effect on perceived ejaculatory control but no direct effect on satisfaction with sexual intercourse or personal distress associated with ejaculation [1]. Nevertheless, there are few psychometrically sound PRO measures that cover the full range of PE problems [2,14,19], although the more recently developed single-item PE measures have evidence supporting reliability and validity [6,15]. More recently, Althof and colleagues have developed and validated the Index of Premature Ejaculation which covers many of the key aspects of the disorder [20].

Given that patient perceptions and the psychological component of PE can be a significant factor in diagnosis and that PROs are important for PE assessment, direct patient input adds critical insight for characterizing the condition and its impacts. However, studies involving open-ended discussions among males with PE and their partners are lacking.

The purpose of this study was (1) to characterize the psychosocial burden of having PE from the perspectives of heterosexual males with PE and female partners of males with PE in the US, UK, Italy, France, Germany, and Poland and (2) to cognitively debrief males with PE and female partners of males with PE about their understanding of the recently developed single item measures of four dimensions of PE. The qualitative research was conducted using focus groups and one-on-one interviews with participants which first examined the burden and problems associated with PE in US participants. In the next study phase, in-person interviews examined the burden associated with PE and cognitively debriefed participants in different countries on the single-item PE measures in European participants. This research was completed before the publication of more recent PE measures, such as the Index of Premature Ejaculation [20] and other scales [19].

## Methods

Fourteen focus groups were convened in the US (eight in Denver, CO, three in Chicago, IL, and three in Atlanta, GA) with heterosexual males with PE and female partners of males with PE to explore the psychosocial impact and burden of PE. Separate groups were conducted for male and female participants. In addition, one-on-one in-depth interviews were conducted with males with PE and female partners of men with PE in the UK, Italy, France, Germany, and Poland (interviews also were conducted with a sample of men in the US). In addition to discussion on the psychosocial impact of PE, the in-depth interviews also included obtaining feedback on a male and female version of a brief PRO measure, the Premature Ejaculation Profile [6,15], which was developed before the focus groups and in-depth interviews and which focuses on control and satisfaction with sexual intercourse.

Study participants (or female partners) met the following criteria: (1) 18–70 years of age; (2) heterosexual; (3) had one sexual partner for past six months; (4) not taking medication that impairs sexual function; (5) experiences PE in greater than 50% of sexual intercourse events based on self-report; and (6) no self-reported problems associated with erectile dysfunction or sexual impotence, low libido, previous pelvic surgery or injury, previous spinal surgery or injury, chronic prostates, or urethritis. For the men, they needed to report that their average IELT was < 2 minutes and that they reported distress associated with their PE. For this study, the female partners of men with PE were not required to be in relationships with the men with PE participating in this study. Heterosexual couples were not recruited, and male or female participants only needed to report that they (for males) or their male partners (for females) experienced PE in the majority of their sexual intercourse events.

The moderators used interview guides that included open-ended questions on the following topics: general comments or concerns regarding PE, development and experience of PE for the male partner, emotional distress and impact of PE, partner communication about PE, PE effect on physical and emotional intimacy with their partners, impact of PE on the overall partner relationship, and characteristics of a successful treatment for PE.

In addition to inquiring about the general impact of PE, the in-depth interviews included administration of the male and female partner versions of the single item PE measures to male and female participants, respectively. Each version contains four items that ask about control over ejaculation, satisfaction with sexual intercourse, personal distress over PE, and relationship difficulty because of PE [6,15]. Responses are captured on five-point Likert-type scales. The PE related items was translated and linguistically validated into the different languages using standardized methods [21,22]. After completing the PE related items, semi-structured cognitive debriefing interviews were conducted. The participants were asked to discuss the meaning of each item, their understanding of the item content, and what they thought about when choosing a response. The interviews were conducted in the native language and were simultaneously transcribed into English.

### Analysis

The objective of the analysis was to review the qualitative data to characterize the functional, emotional, and interpersonal impacts of PE from the patient and partner perspective. A grounded theory approach was applied for the analysis [23,24]. This approach means that themes that are identified need to be grounded or rooted based on examination of the data and not initially imposed on the data. The research team, which included five health services researchers, used Ethnograph qualitative data analysis software, version 5.08 (Qualis Research, Denver, CO), to help organize the data. As is the nature of qualitative research, this was a fluid process; hence, all ideas and comments were met with an open discussion. The researchers convened a series of meetings to identify and finalize the major themes and respective categories within each theme.

The cognitive debriefing interviews for the single item PE measures were content analyzed and summarized to determine the participants' understanding and comprehension of each item and response scale.

## Results

A total of 172 males with PE and 67 female partners of men with PE participated in this study. A total of 51 men and 17 women participated in the US focus groups, and 85 men and 46 women, who were recruited from the UK (22 men; 10 women), Italy (20 men; 10 women), France (20 men; 10 women), Poland (19 men; 10 women), Germany (20 men; 10 women), and the US (19 men only), participated in the one-on-one interviews. An additional 36 men and four women from the European countries provided feedback on the PE measures, but did not participate in the open-ended qualitative component of the interviews. Demographic characteristics are summarized in Table 1 by country and gender. The US study participants were primarily Caucasian (men: 78%; women: 94%). In the European sample, the mean age of the men was 43 years (range 25 to 68) and the mean age for women was 40 years (range 23 to 61), and most were Caucasian (men: 77%; women: 82%). For all the men, most participants (66%) self-reported that their latency time was less than two minutes.

**Table 1:** Demographic characteristics of the US and European participants

	**Country**						
	**United States**		**Poland**	**Germany**	**Italy**	**UK**	**France**
	**FG**	**I**					
N	68	19	29	30	30	32	30

Gender							

Males	75%	100%	66%	67%	67%	69%	67%
Females	25%	0%	34%	33%	33%	31%	33%

Mean Age							

Males	42.1	46.2	44.6	44.2	41.1	37.3	41.8
Females	40.8	--	42.2	46.2	41.7	33.9	34.4

% Caucasian							

Males	78%	84%	100%	95%	100%	55%	45%
Females	94%	--	100%	90%	100%	60%	60%

Marital Status							

Males							

Married	41%	84%	68%	30%	47%	41%	55%
Single	43%	11%	14%	35%	42%	41%	30%
Divorced	16%	5%	16%	22%	11%	9%	0%
Other	0%	0%	2%	13%	0%	9%	15%

Females							

Married	59%	--	80%	10%	50%	10%	30%
Single	41%	--	10%	50%	30%	80%	40%
Divorced	0%	--	0%	30%	20%	0%	10%
Other	0%	--	10%	10%	0%	10%	20%

Abbreviations: FG = focus group; I = individual interview

### PE impact

A common issue arising from review of the focus group and in-depth interview data was that all participants felt that, because of PE, they lacked something that could bring more fulfillment into their lives. Lack of control over ejaculation led to dissatisfaction with sexual intercourse for the males and partners of men with PE and, in many cases, resulted in the disruption of their relationship. Even among those participants who stated that they were sexually satisfied, the male and female participants indicated that they felt something was missing from their relationship, and this affected their sense of intimacy. The participants employed numerous coping strategies to attain sexual satisfaction, and many sought medical or psychological therapy to help address the effects of having PE. However, based on the feedback, it appears that some individuals affected by PE were reluctant to seek help given the stigma associated with this problem.

The major themes raised by the study participants included control over ejaculation, satisfaction with intercourse, emotional impact and distress, relationship problems, and partner reactions (Table 2). These themes as well as the men's coping strategies are discussed below.

**Table 2:** Major themes and respective categories describing PE impacts

**Theme**	**Definition**
CONTROL	Control of ejaculation

Time	Reference to or evaluation of latency time (e.g., wanting to last longer)
Control	Desire to be able to change the timing of ejaculation

SATISFACTION	Satisfaction with intercourse

Unsatisfied with Sex	Feeling dissatisfied with sex
Partner Satisfaction	Desire to please partner during sexual intercourse
Intimacy	Dissatisfied with feeling of closeness associated with sex
Pleasure	Desire to enjoy sexual intercourse

EMOTIONS	Male's feelings associated with PE

Ego	Decreased self-confidence and self-esteem
Embarrassed	Embarrassed due to PE
Expectation	Not meeting social expectations
Inadequate	Feelings of being inferior; not being able to fulfill manly role
Anger	Anger
Anxiety	Worry or anxiety
Disappointment	Disappointed or unhappy
Frustration	Frustrated

RELATIONSHIP	Expressions about relationship related by either male or female partner

Relationship Frustration	Feelings of frustration within the relationship due to PE
Relationship Insecurity	Feeling insecure about relationship; have difficult relationship
Relationship Tension	Feelings of tension with the relationship due to PE

PARTNER REACTIONS	Female partner reactions to PE

Partner Anger	Partner feeling angry or annoyed
Partner Disappointment	Partner expressing feelings of being disappointed or sad
Partner Frustration	Partner frustrated
Partner Reassurance	Partner reassuring male sufferer to minimize the magnitude of problem
Partner Avoid Sex	Partner avoids or has lost interest in sex due to PE
Relief	Desires quick sex because has busy schedule or is tired

#### Control over ejaculation

Control of ejaculation emerged as a central concept and represented an essential component of PE. The absence of ejaculatory control was the principal problem identified by the male and female participants. Participants felt that they did not have control: "Well, for me, I don't feel like I really ever have control"; "If I had the control as to when I did it. That's what I'd want." In response to the question about how to determine whether a treatment for PE would be considered successful, respondents indicated that control of ejaculation was critical: "If it increases – if it doubles it, yeah that's success"; "I feel if I had control, then this wouldn't be an issue at all"; "I mean, the ultimate success would be unlimited control."

**Figure 1:**
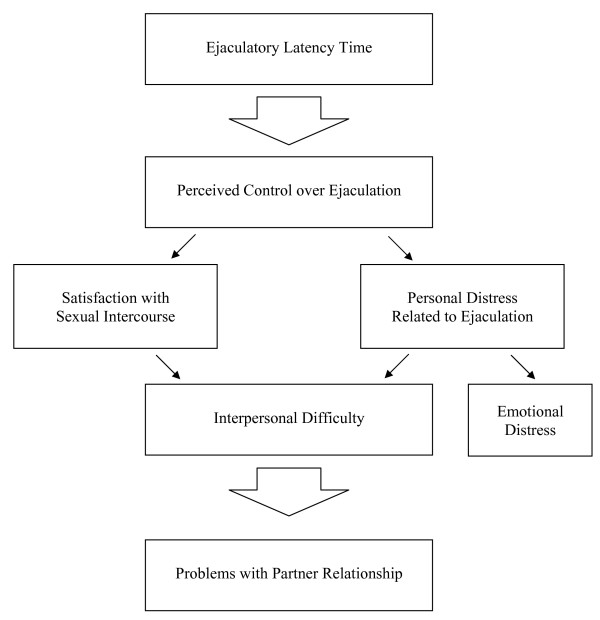
Association among premature ejaculation-related patient outcomes.

Latency time was directly connected with the men's viewpoint about control of ejaculation. The experience of PE was essentially based on lack of control over ejaculation and the other related PE problems were directly associated with ejaculatory control. Figure 1 summarizes the association between PE related perceptions based on the discussions and interviews with men with PE and female partners of men with PE. Perceived lack of ejaculatory control was related to levels of satisfaction with sexual intercourse and personal distress associated with the sexual act. These problems results in interpersonal difficulty and problems with intimacy between the male and his partner, and increased emotional distress among the men and to some extent to women in this study. Finally, problems with interpersonal relations and intimacy resulted in broader problems in their relationships.

#### Satisfaction with sexual activities

Satisfaction with sexual intercourse was another important concept, and the perception of satisfaction with the sex act was directly associated with the sense of ejaculatory control. For the men, their level of satisfaction was directly linked to their partner's satisfaction with sexual intercourse. A common comment was: "That's the goal, is for both of us to be satisfied". However, even when men discovered ways for their partners to attain orgasm, they still felt that PE affected both their own and their partner's overall satisfaction. This concept is illustrated by the following: "Orgasm isn't what she's looking for. She's looking for the whole piece"; "But even when she's completely happy, I feel like I'm still not lasting as long as I should"; "I'd say sex is generally satisfying, but I think it could be better"; and "I mean, it's satisfying, but maybe it's, you know, not as much so."

The participants felt that they were missing out on an important factor in feeling close to their partners because of their lack of control over ejaculation. The male participants stated that: "It definitely takes the intimacy out, and it just becomes physical at that point"; "I would say it affects my love life, not my sex life so much"; "It prevents that closeness from being, you know, fully consummated"; and "I'm looking for the intimacy that we lost." The impact of PE on this sense of intimacy and closeness was important to men even if their partners lose interest in the problem. Many of the male participants in long-term relationships have learned to adjust to the problem but they still felt unfulfilled: "She really never complains, but I felt it myself"; "It's time to ...try and fix it; She seems to really enjoy sex, and I'd like to have that same degree of enjoyment"; "I think, if she was honest herself, she would as well [believe that PE was a problem]"; and "It's something that's very important to me, and in the case of my wife, it's not important to her anymore."

Both men and women expressed that they sometimes felt that initiating sex was not worthwhile because they would not achieve sexual satisfaction and they did not want to feel disappointed or cause their partners to feel disappointed. The female participants reported that their male partners reduced the number of sexual advancements due to fear and anxiety related to PE. The Polish women specifically reported feelings of low self-worth and unattractiveness due to the lack of sexual advancements from their partners. Some women in the UK and Poland reported finding sexual satisfaction with others because of their partner's PE.

#### Emotional well-being and self-confidence

Many of the men reported significant emotional distress associated with their sexual dysfunction. PE affected them emotionally and clearly impacted their self-confidence: "Your lack of control there in that situation makes you feel inferior"; and "When you can't satisfy your woman, you somehow feel like there's a large part of you that is missing or failed." In addition to inadequacy, many of the men reported feeling anxious, frustrated, angry, and disappointed. The men felt frustrated about their PE and how it affected their intimacy with their partners and sexual relationship. In general, the emotional impacts were associated with not feeling manly as defined by societal expectations. Common statements from men were: "And it all links to not having control. You're angry because you can't control the situation"; and "I would like to be able to enjoy the whole sexual experience without having an uptight feeling."

The women perceived their male partner's emotional distress but some noted that this made it difficult to address the problem. Illustrative comments from women included: "I realize I can't say anything because he's feeling inadequate"; "I don't want to bring it up, because I don't want to affect his ego"; and "You don't want to say too much because you don't want to offend them." In general, the women perceived anxiety, reluctance, and avoidance from their partners regarding sex. A predominant perception among the men across different countries was that their partners were reluctant to discuss PE. Alternatively, several of the women indicated that their partners were in denial and would not discuss problems associated with PE: "He's like, we don't have that problem"; and "Because he doesn't think it's big deal, and he doesn't – that annoys me." Women in the US, Italy, and Poland mentioned that their partners were most often in denial about their PE and would not admit that there was a problem at all.

Some men indicated that they found it very difficult to raise the topic of PE, and it was not easy for some of the men to volunteer for the study: "It's generally not something I talk about"; "I needed a little bit of courage to come in." Many of the female participants indicated that their partners would be too self-conscious to seek help: "He wouldn't be able to take it"; and "I asked him to come here, and he says 'there's no way I could do that."' Many of the men did indicate though that they tried to seek help in various ways, including buying relevant books, seeing a psychiatrist, going to hypnotherapy, and discussion with their doctors. Some struggled with whether or not they should see a psychologist versus a medical doctor, and many indicated that "It's hard to find information on this kind of stuff."

#### Relationship with partner

The negative effects of PE had profound impacts on the relationship between the men and their partners. For many men "You feel like you're not connecting in your relationship." In some cases, issues with PE led to concerns about the stability of the relationship: "I kind of question, you know, how good the relationship is when my girlfriend says, you know, this is not going to continue... this relationship is over if things don't improve." Men in long-term relationships generally had less anxiety about PE and reported better communication with their partners about PE. However, among women in long-term relationships, only those in Germany and France reported minimal anxiety and good communication about PE. Many younger male respondents reported feeling that their relationship could be in jeopardy, and specifically some males in the UK, Italy, Germany, and France were worried about losing their partners because of PE: "She said this relationship is going to be over if I don't do something about it".

Most women expressed disappointment, dissatisfaction and frustration in their sexual relationship and level of intimacy because of PE: "So then there's just kind of a disappointment." Some expressed anger, particularly when their partners denied that there was a problem: "You feel like you don't get his attention or anything." Many of the women were reassuring, which helped the males feel less anxious about sex. However, many of the men noted that they felt this reassurance was not always genuine; they sensed that the women did not want to undermine their self-confidence: "I think she's being sensitive not to shatter my precious male ego." One reaction identified only among some female partners was a feeling of relief because, given that they had busy schedules, they did not have time for lengthy sex.

#### Coping strategies

The men employed various physical and mental strategies to try and deal with their PE. These strategies included trying to detach themselves and think of other things during sex, using humor to decrease the embarrassment of PE, pacing themselves during sex, and interrupting intercourse to prolong latency time and to improve ejaculatory control. For some, it was helpful to discuss their problems with others, including their partners or physicians. In general, these coping strategies were perceived to have varied success and the underlying problems associated with PE remained.

### Feedback on PE measures

The cognitive debriefing interviews on the male and female versions of the PE measures substantiated the relevance of control of ejaculation, satisfaction with intercourse, personal distress, and relationship difficulty among the participants. The participants believed that the PE measures focus on important consequences of PE, and they did not have difficulty understanding the item content or in responding to the questionnaire. Control over ejaculation was described by males as being able to stop or hold back ejaculation if desired and the time to ejaculation. Women uniformly reported this item was associated with time to ejaculation. In some cases, men and women included in their descriptions of control the ability of the man to prevent ejaculation until his partner attained orgasm.

In response to the satisfaction with sexual intercourse item, most of the men thought about the pleasure they experience during sexual intercourse, and this was directly related to the ability of bringing their partners to climax during vaginal intercourse. The women reported that the meaning of this item was based on their ability to achieve orgasm through vaginal penetration and sexual intercourse. Some respondents also indicated that their satisfaction was related to the entire sexual encounter including both the physical and emotional response.

For the item on personal distress related to PE, all participants understood distress to be negative emotions resulting from PE although there was variation in how "distress" was defined. Distress was generally interpreted to include a range of possible emotions, including feeling inadequate, disappointed, annoyed, like a failure, frustrated, and anxious. The women defined "distress" as sexual frustration, irritation, sexual dissatisfaction, or decreased sexual interest. Some variation was observed among countries with respect to interpreting degrees of distress. Specifically, in the US sample, "distress" was understood in levels of severity. In Germany, a few participants did not attach different levels of severity to "distress" – one was either distressed or not distressed. In France, most participants identified "distress" as feeling upset.

The "relationship difficulty due to PE" item generally was interpreted similarly across participants. The majority of participants explained that this item referred to problems in their partner relationships because of PE. These problems included being disappointed in the relationship, experiencing tension, being frustrated or sensing frustration in a partner, arguing, and disruption in the stability of the relationship. In responding to this item, some participants made a distinction between their sexual relationship and their overall relationship. For example, some participants in long term relationships, or who were older, focused on their sexual relationship when responding. In contrast, younger males or those in shorter relationships expressed focused on their overall relationship when responding. Most French participants thought about this item in terms of the effects on their sexual relationship versus the effects on their overall relationship.

## Discussion

The findings from this study suggest that there is a substantial psychosocial burden associated with PE on heterosexual males and partners of men with PE, and that the major impacts appear to be consistent in the US and Europe. The findings identified control of ejaculation as a central concept for PE. The absence of ejaculatory control results in dissatisfaction with sexual intercourse and personal distress. The level of satisfaction with sexual activities and personal distress associated with PE, leads to increased general emotional distress and relationship problems. Even in relationships where the participants noted that they were generally satisfied with sexual activities, both males and females indicated that PE was keeping them from feeling fulfilled or attaining complete intimacy in their relationship.

This multi-country study, which included both males with PE and female partners of males with PE, expands on previous qualitative research conducted by Symonds and colleagues in a US-based study of 28 males with PE [12]. Consistent with the Symonds study, this study found PE to have detrimental effects on self-confidence and partner relationships and that men with PE experience anxiety, embarrassment, and a lack of fulfillment. Because this study included female partners of men with PE, although not necessarily the partners of the male participants, we were able to more thoroughly explore the impact of PE on the female partner and on the relationship dynamic. Clearly, in many cases both men and women have difficulty discussing PE and therefore the effects reported here may be even more substantial than those reported in this study. The study participants indicated that many individuals experiencing PE are unlikely to agree to participate in qualitative research.

The results further confirm the central role of ejaculatory control in understanding the impact of PE on men and their partners. Latency time and the perception of control over ejaculation affect the men's satisfaction with sexual intercourse and their distress related to PE. The qualitative research is consistent with the findings of a large study of men with PE [1,6] and other studies [19,20]. Patrick and colleagues found that IELT was not directly associated with satisfaction with sexual intercourse or personal distress but that this relationship was mediated by perceptions of ejaculatory control [1]. Personal distress and satisfaction with sexual intercourse were directly associated with the interpersonal relationship between the male and his partner.

Some researchers question the need for patient reported measures for PE outcomes assessment [4,5]. The current study findings suggest that more complete understanding of PE and the effectiveness of PE related treatments depend on assessing both IELT and patient perceptions about control of ejaculation, personal distress and satisfaction with sexual intercourse. More recently, several patient-reported measures of PE have been developed covering these concepts, although psychometric attributes vary among different scales [19].

Based on the discussion by participants, sexual satisfaction for men is strongly influenced by their female partner's report of satisfaction with sexual activities. The men reported feeling anxiety and reluctance about sex for fear of disappointing their partner. Men also felt like failures because of their inability to affect their ejaculatory control.

The cumulative effects of PE disrupt the partner relationship, causing instability. Female partners of men with PE react in various ways, and many experience disappointment and frustration. In some cases, the women in this study were concerned about damaging their partner's ego and self-esteem so they avoided direct communication. Communication was a significant issue between couples and was associated with age and length of relationship. In general, couples in longer and stable relationships found ways to communicate about and adapt to the male's PE. For younger men and those in shorter relationships, the extent and nature of the communication between partners was an important driver in perceptions of personal and relationship difficulty.

Consistent to the finding by Symonds and colleagues [12], many of the men in this study noted using a number of behavioral/psychological strategies to handle their PE [12]. The men sought help in various ways, including purchasing books and visiting a therapist or physician. However, these interventions varied in effectiveness in treating their PE.

The participants confirmed that the four single-item PE measures were relevant for capturing the major impacts of PE. These findings are consistent with newer patient-reported PE outcome measures [20]. The participants thought that the content and response scales were understandable and clear. However, there was some variation between countries in views about rating distress, with some participants preferring simple distressed/not distressed ratings while the majority thought that rating levels of severity of distress was best. In future research studies of men with PE, this research suggests that these PE-related measures should be supplemented with other PRO measures to capture effects on the partner relationship and general psychological well-being. Symonds et al. also emphasized the need for assessments to include PRO domains such as impact on current relationship, psychological well-being, sense of masculinity and impact on the partner [12].

Limitations of this study are primarily associated with the generalizability of the findings to the larger PE population. First, this study relied on self-report to identify subjects with PE. Research has indicated that subjective self-reports of latency time may be inconsistent with prospective latency time measurement [6,25,26]. Thus, to the extent that men with self-reported PE differ from those with clinician-diagnosed PE, the findings may not be generalizable to all men with PE. Second, the participants were limited to heterosexuals and those who were in stable relationships and, therefore, important concepts that may affect other patient groups may have been missed. Third, we included only those who felt comfortable discussing their own or their partner's condition. Finally, we did not recruit male and female partner pairs, but focused on identifying men with PE and female partners of men with PE. Future research is needed on men with PE and their actual partners to confirm and extend these results. Nevertheless, we believe that the findings from this study provide a good framework for understanding the impact of PE from the perspective of males with PE and of female partners of men with PE. A key strength of this qualitative research study is the large number of male and female participants and the representation of participants from the US and several European countries.

## Conclusion

This in-depth qualitative study provides valuable insight on the substantial psychosocial burden of PE in the US, UK, Germany, Italy, France, and Poland. The concept of ejaculatory control was central for understanding the effects of PE on men and their partners, with consequent impact on satisfaction with sexual intercourse and personal distress. PE was associated with wide-ranging impacts for both men with PE and their partners. This study confirms the importance of patient perceived aspects of PE and demonstrates that the single-item PE measures cover the key and relevant content for men with PE and their partners. Further, the qualitative data collected in this study can help inform the content of new and expanded PRO measures for use in PE research.

## Abbreviations

PE: Premature ejaculation; DSM-IV-TR: Diagnostic and Statistical Manual of Mental Disorders; PRO: Patient-reported outcomes; IELT: Intravaginal ejaculatory latency time

## Competing interests

Dr. Revicki, Ms Howard, Hanlon and Mannix are consultants and at the time the work was performed were all employed by United BioSource Corporation. This project was funded by Johnson & Johnson Pharmaceuticals under the direct of Dr. Rothman and Ms Greene.

## Authors' contributions

DR, KH, SM and JH designed the qualitative research study, completed the content analyses, interpreted the qualitative data, and drafted and revised the manuscript. KH, SM and JH conducted the qualitative research and analyzed the data. MR and AG conceptualized and designed the study, interpreted the qualitative data and drafted and revised the manuscript. All authors reviewed and approved the final version of this manuscript.
